# Predictive factors for persistent postoperative hydrocephalus in children undergoing surgical resection of periventricular tumors

**DOI:** 10.3389/fneur.2023.1136840

**Published:** 2023-07-04

**Authors:** Zitao Chen, Ming Zhou, Huantao Wen, Qiang Wang, Jianwei Guan, Yuan Zhang, Wangming Zhang

**Affiliations:** The National Key Clinical Specialty, The Engineering Technology Research Center of Education Ministry of China, Guangdong Provincial Key Laboratory on Brain Function Repair and Regeneration, Department of Neurosurgery, Zhujiang Hospital, Southern Medical University, Guangzhou, China

**Keywords:** hydrocephalus, risk factors, CSF management, pediatric brain tumors, surgical treatment

## Abstract

**Objective:**

The aim of this study is to identify the factors predicting persistent hydrocephalus after periventricular tumor resection in children and assess the need and efficacy of perioperative cerebrospinal fluid (CSF) intervention.

**Methods:**

We performed a retrospective analysis of pediatric patients who underwent resection surgery of a periventricular tumor between March 2012 and July 2021 at the Department of Neurosurgery in Zhujiang Hospital of South Medical University. Demographic, radiographic, perioperative, and dispositional data were analyzed using univariate and multivariate models.

**Results:**

A total of 117 patients were enrolled in our study. Incidence of postoperative persistent hydrocephalus varied with tumor pathology (*p* = 0.041), tumor location (*p* = 0.046), surgical approach (*p* = 0.013), extension of resection (*p* = 0.043), tumor volume (*p* = 0.041), preoperative Evan's index (*p* = 0.002), and preoperative CSF diversion (*p* = 0.024). On logistic regression, posterior median approach (OR = 5.315), partial resection (OR = 20.984), volume > 90cm^3^ (OR = 5.768), and no preoperative CSF diversion (OR = 3.661) were independent predictors of postoperative persistent hydrocephalus. Preoperative Evan's index is significantly correlated with tumor volume (*p* = 0.019). Meanwhile, the need for preoperative CSF drainage in patients in this cohort was significantly correlated with tumor location (*p* = 0.019).

**Conclusion:**

Tumor pathology, location, surgical approach, the extension of resection, tumor volume, preoperative Evan's index, and preoperative CSF diversion were considered to be predictive factors for postoperative persistent hydrocephalus. Notably, posterior median approach, partial resection, and tumor volume > 90cm^3^, without preoperative CSF diversion, were identified as independent risk factors for persistent postoperative hydrocephalus. Preoperative identification of children at risk of developing persistent postoperative hydrocephalus would avoid delays in planning the cerebrospinal fluid diversion. Active and effective preoperative hydrocephalus intervention in children with periventricular tumors is beneficial to reduce the incidence of persistent hydrocephalus and ventriculoperitoneal shunt surgery after resection.

## Introduction

Pediatric central nervous system (CNS) tumors are the second most common childhood malignancy and the most common solid tumors in children. In addition, according to surveillance, brain tumors are the most common cause of death among all childhood cancers (1). In children and adolescents, the incidence rate of primary CNS malignant or non-malignant tumors in the United States was 6.14 per 100,000 between 2013 and 2017 (2). The signs and symptoms in children's brain tumors depend on various factors, including the location of the tumor, the age of the child, the rate of tumor growth, and so on. Hydrocephalus caused by the central nervous system, defined as tumor-associated hydrocephalus, may occur in more than 50% of brain tumors in children (3) and have a higher occurrence in periventricular tumors. It can result in high intracranial pressure and related symptoms such as headache, vomiting, even unconsciousness, and long-term cognitive or adaptive deficits in children's brain development. Tumor-associated hydrocephalus is mainly an obstructive type, rarely communicating, or hypersecretory type (in the case of plexus papilloma) (4). Brainstem gliomas and other third ventricle tumors can present with aqueduct obstruction and new-onset hydrocephalus, while the most common pediatric posterior fossa brain tumors, including cerebellar astrocytoma, medulloblastoma, and ependymoma, often manifested as hydrocephalus, are caused by the fourth ventricle outlet obstruction (5). Most cases of hydrocephalus associated with pediatric brain tumors can be successfully cured after surgical resection; however, in 10–40% of cases, it can persist after surgery requiring temporary or permanent cerebrospinal fluid drainage, such as an external ventricular drain (EVD) or a ventricular–peritoneal shunt (VPS) (6, 7). Previous studies by Riva-Cambrin (8) and Foreman (9) have shown that young age, moderate-to-severe hydrocephalus, transependymal edema, presence of cerebral metastases, and tumor pathology (medulloblastoma and ependymoma) on presentation predict postoperative persistent hydrocephalus. However, it was refuted by other researchers and remains controversial. An effective predictive model for persistent postoperative hydrocephalus has not yet been established (7), which makes it difficult for neurosurgeons to accurately assess the occurrence of persistent hydrocephalus after tumor resection and appropriately start timely cerebrospinal fluid management. The timing of tumor-associated hydrocephalus management in children is the subject of debate. Preoperative temporary external ventricular drainage (EVD) placement has been reported to add the advantage of being easy to remove after surgery as the majority of patients do not develop persistent postoperative hydrocephalus (10). Proponents of permanent preoperative cerebrospinal fluid diversion (VPS or ETV) argue that preoperative shunt reduces the technical difficulty of resection surgery and improves postoperative course and overall mortality. Although several studies have compared the efficacy and safety of VPS and ETV in pediatric postoperative persistent hydrocephalus, there is still no consensus on which method is preferable for the treatment of tumor-associated hydrocephalus (7, 11).

In the current study, the type of treatment indicated is directed by the cause of hydrocephalus (communicating or non-communicating). For example, if the cause of the hydrocephalus is eliminated, it may no longer need to be shunted. In cases of childhood brain tumors, surgical evacuation might be an appropriate option in combination with or without shunting (12). The main controversy is whether to treat hydrocephalus before tumor resection, and some authors advocate preoperative shunts while others recommend EVD (13). We aimed to seek factors, which might be correlated with the development of persistent hydrocephalus following the resection of pediatric brain tumors to evaluate the indication for CSF drainage before surgery.

## Materials and methods

This study was approved by the Zhujiang Hospital Ethics Committee, which determined consent was not required owing to the study's retrospective nature. This research conforms with the Declaration of Helsinki. The treatments provided were based on the guidelines of the Zhujiang Hospital. Data in this study will be made available upon reasonable request.

### Study design

This retrospective descriptive cohort investigated the incidence of persistent postoperative hydrocephalus and its causative factors in a consecutive group of 117 patients who underwent surgery for periventricular tumors between March 2012 and July 2021 at the Department of Neurosurgery in Zhujiang Hospital of South Medical University.

The inclusion criteria were as follows: (1) age less than 15 years old; (2) single intracranial neoplasm detected by preoperative magnetic resonance imaging; (3) with periventricular tumor-associated hydrocephalus before surgery; (4) surgical resection of the lesion; and (5) a tumor confirmed by pathological diagnosis according to the 2016 WHO classification (14). All tumors presented with hydrocephalus at diagnosis and extended beyond the boundaries of the ventricles (lateral, third, or fourth ventricle).

Patients were excluded if (1) evidence of multiple tumors or repeated surgery for tumor; (2) with other central nervous system diseases or lesions such as hemorrhage, injury, arteriovenous malformation, meningitis, or history of craniocerebral surgery before admission; and (3) loss of necessary clinical or image data. One hundred and seventeen patients with tumor-associated hydrocephalus were identified.

Evan's index (EI) is defined as the maximum between the frontal horns divided by the maximal width of the inner table (15). In clinical practice, Evan's index is often used to define the severity of hydrocephalus. EI between 0.28 and 0.34 is mild hydrocephalus, between 0.34 and 0.40 is moderate hydrocephalus, and larger than 0.4 is severe hydrocephalus.

**Figure 1:**
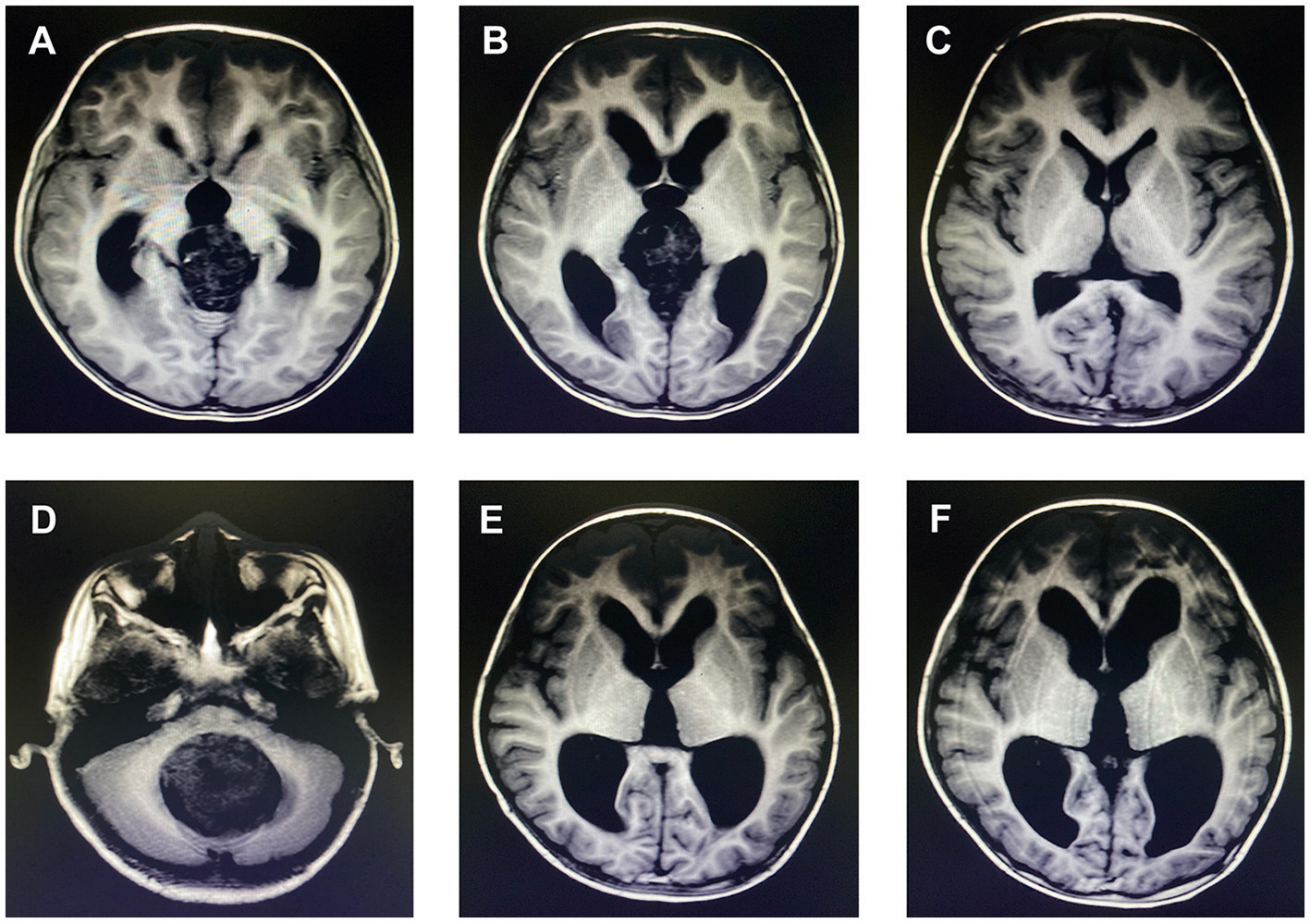
Patient 1. male, 3-year-old, immature teratoma in the third ventricle, volume: 3.8×3.2×3.5 cm, underwent preoperative Ommaya reservoir placement and then gross total resection. No symptoms and signs of increased intracranial pressure after surgery. **(A)** Imaging manifestation of tumor from preoperative MRI. **(B)** Imaging manifestation of enlarged lateral ventricle from preoperative MRI. **(C)** Imaging manifestation of lateral ventricle from MRI 3 months after tumor resection, it showed the enlarged ventricle returned to normal size. Patient 2. female, 7-year-old, medulloblastoma in the fourth ventricle, volume: 5.0 × 4.9 × 5.2 cm, underwent preoperative EVD and then gross total resection. Symptoms like headache, vomiting, ataxia were seen after surgery. **(D)** Imaging manifestation of tumor from preoperative MRI. **(E)** Imaging manifestation of enlarged lateral ventricle from preoperative MRI. **(F)** Imaging manifestation of lateral ventricle from MRI 3 months after tumor resection, it showed the persistent enlarged ventricle.

The persistent postoperative hydrocephalus in this study was defined as: (1) Symptoms of intracranial hypertension continue after tumor resection and CSF diversion, such as headache, vomiting, ataxia, and so on. (2) Postoperative magnetic resonance imaging or CT scan suggests that the expansion of the ventricles reaches the imaging diagnostic criteria of hydrocephalus and the duration is more than 6 months (Figures 1A–F).

We collected the following characteristics: (1) age at diagnosis; (2) tumor site, size, and histology; (3) preoperative Evan's index; (4) extension of surgical resection (gross total, subtotal, partial, biopsy); (5) intracranial infection or hemorrhage after surgery; (6) placement of EVD, VPS, ETV, or Ommaya reservoir before or after surgery and related complications; (7) presence/absence of postoperative hydrocephalus; (8) follow-up. The availability of paired preoperative and postoperative images (CT scans or MR) was reviewed and unavailability led to exclusion analysis.

### Statistical analysis

Data analysis appropriate to a cross-sectional study was performed using IBM SPSS version 26 software (IBM Corporation, Armonk, New York, USA). Categorical variables were reported as counts (%), and continuous variables were expressed as mean ± standard deviation or median. The chi-square test and Fisher exact tests were used where appropriate for categorical variables, while Student's *t*-tests and Mann–Whitney *U* tests were used for continuous variables. A simple logistic regression was performed to evaluate factors associated with the persistence of postoperative hydrocephalus. A *p*-value of < 0.05 was considered significant.

## Results

### The baseline of characteristics of pediatric patients

In total, 117 patients (77 male and 40 female children) were included in this study. The median age at the time of surgery was 6.57 years old. Histologically, the most common tumor was medulloblastoma (38.5%), followed by astrocytoma (18.8%) and ependymoma (11.1%). Rare lesions included teratoma, ganglioglioma or oligodendroglioma, craniopharyngioma, germinoma, AT/RT, choroid plexus papilloma, and glioblastoma. The fourth ventricle was the most frequent site of tumors, observed in 50.4% of the cases, followed by cerebellopontine angle (14.5%), third ventricle/pineal gland (14.5%), lateral ventricle (6.8%), cerebral hemisphere (6.0%), thalamus/basal ganglia (4.3%), and sellar region (3.4%). All tumors presented at diagnosis with hydrocephalus and extended beyond the boundaries of the ventricles (lateral, third, or fourth ventricle). Complete resection was achieved in 96 patients (82.1%), subtotal resection in 7 patients (6.0%), partial resection in 12 patients (10.3%), and biopsy only in 2 patients (1.7%). Details of sex, preoperative Evan's index, surgical approach, and tumor volume are shown in Table 1.

**Table 1:** Patients' characteristics and details of tumor.

**Variables**	**Value**
**Sex**	
Female	40 (34.2%)
Male	77 (65.8%)
Age (years)	6.57 ± 4.02
< 3	25 (21.4%)
3–6	30 (25.6%)
6–10	31 (26.5%)
10–14	31 (26.5%)
**Tumor pathology**	
Medulloblastoma	45 (38.5%)
Ependymoma	13 (11.1%)
Astrocytoma	22 (18.8%)
Terotoma	7 (6.0%)
Germinoma	5 (4.3%)
Choroid plexus papilloma	4 (3.4%)
Craniopharyngionma	6 (5.1%)
Ganglioglioma or Oligodendroglioma	7 (6.0%)
Glioblastoma	2 (1.7%)
AT/RT	6 (5.1%)
**Tumor location**	
Fourth ventricle	59 (50.4%)
Cerebellopontine angle	17 (14.5%)
Third ventricle/pineal gland	17 (14.5%)
Lateral ventricle	8 (6.8%)
Thalamus-basal ganglia	5 (4.3%)
Sella	4 (3.4%)
Cerebral hemisphere	7 (6.0%)
**Surgical approach**	
Posterior median	72 (61.5%)
Suboccpital retrosigmoid	5 (4.3%)
Poppen	6 (5.1%)
Triangular	5 (4.3%)
Pterional	11 (9.4%)
Longitudinal fissure	5 (4.3%)
Frontal-parietal valve	10 (8.5%)
Temporo-parietal valve	2 (1.7%)
Ventriculoscope biopsy	1 (0.9%)
**Tumor resection degree**	
Gross total	96 (82.1%)
Subtotal	7 (6.0%)
Partial	12 (10.3%)
Biopsy	2 (1.7%)
**Tumor volume (cm** ^3^ **)**	
< 90	103 (88.0%)
>90	14 (12.0%)
**Preoperative Evan's Index**	
0.27–0.34 (mild) (c)	47 (40.2%)
0.34–0.4 (moderate) (d)	40 (34.2%)
>0.4(severe)(e)	30 (25.6%)
**Preoperative CSF diversion (EVD/VPS/Ommaya reservoir)**	
Yes	66 (56.4%)
No	51 (43.6%)
**Postoperative CSF diversion (EVD/VPS/ETV)**	
Yes	30 (25.6%)
No	87 (74.4%)
Length of stay (days)	30.97 ± 20.95
≤ 21 days (3 weeks)	44 (37.6%)
>21 days (3 weeks)	73 (62.4%)

Values are reported as number (percentage) or mean ± SD. AT/RT, Atypical teratoid/rhabdoid tumor; EVD, external ventricular drain; VPS, ventricular-peritoneal shunt; EVD, endoscopic of ventriculostomy.

Considering CSF management before and after surgery (either temporary or permanent CSF diversion procedure was included), 66 patients (56.4%) underwent preoperative CSF diversion, which included 29 EVDs, 33 Ommaya reservoir placements, and three VPSs. Thirty-one patients (26.5%) underwent postoperative CSF diversion, which included seven EVDs, one ETVs, and 23 VPSs. In general, 69 patients (59.0%) suffered persistent hydrocephalus after tumor resection with or without perioperative CSF management, while 48 patients (41.0%) did not develop this long-term complication (Table 1). In particular, 50% (33/66) of patients, who underwent prophylactic CSF diversion (temporary or permanent) before resection, developed persistent hydrocephalus 6 months after surgery. In contrast, 70.6% (36/51) of patients, who did not have prophylactic CSF diversion, suffered persistent hydrocephalus after surgery (Table 2).

**Table 2:** Univariate analysis of the association between factors and persistent postoperative hydrocephalus.

**Variables**	**Postoperative Hydrocephalus**		*P*-value^*^
	**Yes (*****n** =* **69)**	**No (*****n** =* **48)**	
**Sex**			
Female	26 (37.7%)	14 (29.2%)	0.340^‡^
Male	43 (62.3%)	34 (70.8%)	
**Age (years)**	**9.1** ±**3.3**	**8.9** ±**3.9**	**0.481** ^§^
< =3	15 (21.7%)	10 (20.8%)	0.906^‡^
>3	54 (78.2%)	38 (79.2%)	
**Tumor pathology**			**0.041** ^*^^†^
Medulloblastoma	29 (42%)	16 (33.3%)	
Ependymoma	5 (7.2%)	8 (16.7%)	
Astrocytoma	12 (17.4%)	10 (20.8%)	
Terotoma	1 (1.4%)	6 (12.5%)	
Germinoma	2 (2.9%)	3 (6.3%)	
Choroid plexus papilloma	3 (4.3%)	1 (2.1%)	
Craniopharyngionma	5 (7.2%)	1 (2.1%)	
Ganglioglioma or Oligodendroglioma	4 (5.8%)	3 (6.3%)	
Glioblastoma AT/RT	2 (2.9%) 6 (100%)	0 (-) 0 (-)	
**Tumor site**			**0.046** ^*^^†^
Fourth ventricle	39 (56.5%)	20 (41.7%)	
Cerebellopontine angle	5 (7.2%)	12 (25%)	
Third ventricle/pineal gland	8 (11.6%)	9 (18.8%)	
Lateral ventricle	4 (5.8%)	4 (8.3%)	
Thalamus-basal ganglia	4 (5.8%)	1 (2.1%)	
Sella	4 (5.8%)	0 (-)	
Cerebral hemisphere	5 (7.2%)	2 (4.2%)	
**Surgical approach**			**0.013** ^*^^†^
Posterior median	44 (63.8%)	28 (58.3%)	
Suboccpital retrosigmoid	1 (1.4%)	4 (8.3%)	
Poppen	1 (1.4%)	5 (10.4%)	
Triangle	5 (7.2%)	0 (-)	
Pterion	9 (13.0%)	2 (4.2%)	
Longitudinal fissure	4 (5.8%)	1 (2.1%)	
Frontal-parietal valve	4 (5.8%)	6 (12.5%)	
Temporo-parietal valve	1 (1.4%)	1 (2.1%)	
Ventriculoscope biopsy	0 (-)	1 (2.1%)	
**Extension of resection**			**0.043** ^*^^†^
Gross total	52 (75.4%)	44 (91.7%)	
Subtotal	11 (15.9%)	1 (2.1%)	
partial	5 (7.2%)	2 (4.1%)	
Biopsy	1 (1.4%)	1 (2.1%)	
**Tumor volume (cm** ^3^ **)**			**0.041** ^*^^†^
< 90	57 (82.6%)	46 (95.8%)	
>90	12 (17.4%)	2 (4.2%)	
**Preoperative Evan's Index**			**0.002** ^*^^‡^
0.27–0.34(mild)	19 (27.5%)	28 (58.3%)	
0.34–0.4(moderate)	26 (37.7%)	14 (29.2%)	
>0.4(severe)	24 (34.8%)	6 (12.5%)	
**Preoperative CSF diversion (EVD/VPS/Ommaya reservoir)**			**0.037** ^*^^‡^
Yes	33 (47.8%)	33 (68.8%)	
No	36 (52.2%)	15 (31.2%)	
Within “Yes”			0.479^†^
EVD	12 (17.4%)	17 (35.4%)	
VPS	2 (2.9%)	1 (2.1%)	
Ommaya reservoir	18 (26.1%)	15 (31.3%)	
**Postoperative compilcation**			0.167^†^
hemorrhage	5 (100%)	2 (40%)	
infection	0 (-)	3 (60%)	
**Postoperative CSF diversion (EVD/VPS/ETV)**			0.528^‡^
Yes	17 (24.3%)	14 (29.8%)	
No	53 (75.7%)	33 (70.2%)	
Within “Yes”			0.412^†^
EVD	5 (29.4%)	2 (14.3%)	
VPS	11 (64.7%)	12 (85.7%)	
ETV	1 (5.9%)	0 (-)	
**Length of stay**			0.713^‡^
< 3 weeks	25 (36.2%)	19 (39.6%)	
>3 weeks	44 (63.8%)	29 (60.4%)	

Values are reported as number (percentage) or mean ± SD. AT/RT, Atypical teratoid/rhabdoid tumor; EVD, external ventricular drain; VPS, ventricular-peritoneal shunt; EVD, endoscopic of ventriculostomy. ^*^Bold values are significant. ^†^Fisher exact test. ^‡^Pearson χ^2^. ^§^Student t test.

### Univariate analysis of the association between factors and postoperative hydrocephalus

**Figure 2:**
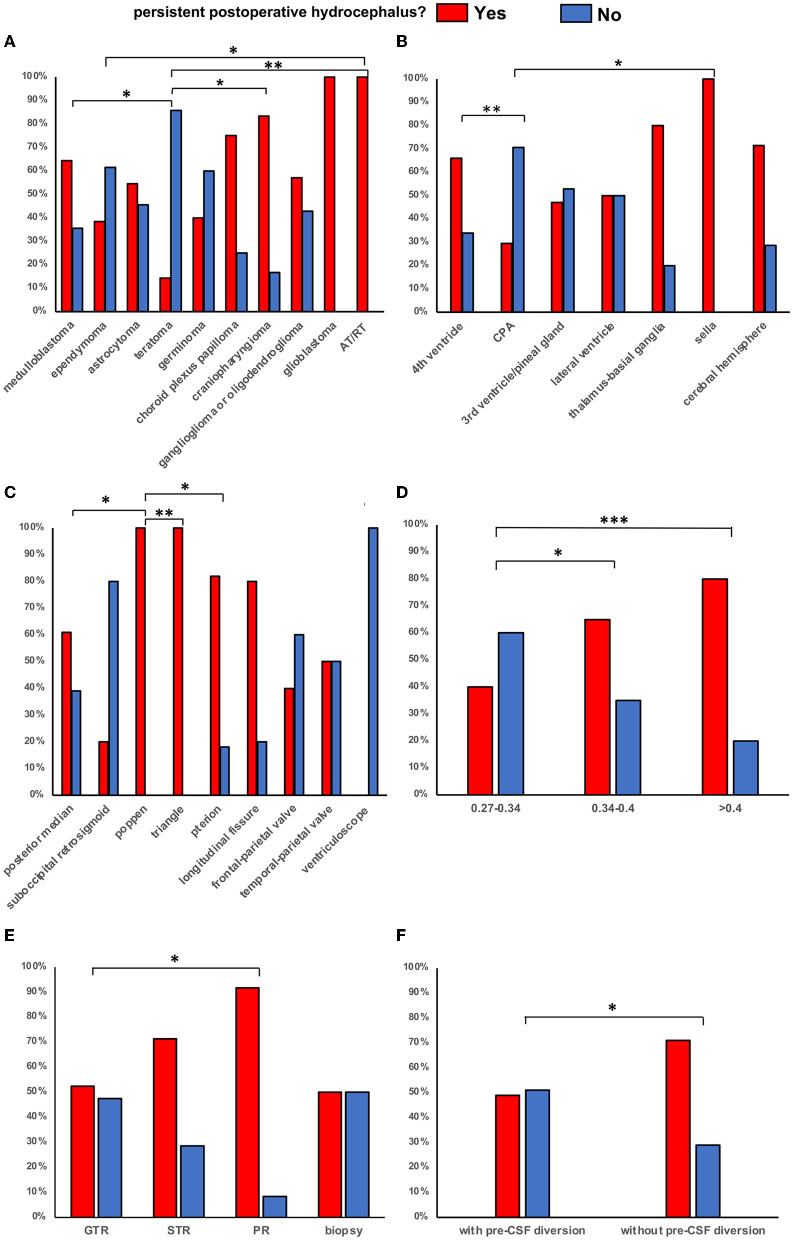
Percentage of patients with and without persistent postoperative hydrocephalus in different tumor pathology, location, surgical approach, preoperative Evan's index, extension of resection and preoperative CSF diversion status. (^*^p < 0.05, ^**^p < 0.01, ^***^p < 0.001. **(A)** Percentage of patients with and without persistent postoperative hydrocephalus in different tumor pathology. **(B)** Percentage of patients with and without persistent postoperative hydrocephalus in different tumor locations. **(C)** Percentage of patients with and without persistent postoperative hydrocephalus in different surgical approaches. **(D)** Percentage of patients with and without persistent postoperative hydrocephalus in different preoperative Evan's index. **(E)** Percentage of patients with and without persistent postoperative hydrocephalus in different extension of resection. **(F)** Percentage of patients with and without persistent postoperative hydrocephalus in different preoperative CSF diversion status.

In the univariate analysis (Table 2), the occurrence of persistent hydrocephalus was significantly correlated with tumor pathology *(p* = 0.041) (Figure 2A), tumor location (*p* = 0.046) (Figure 2B), surgical approach (*p* = 0.013) (Figure 2B), tumor volume (*p* = 0.041) (Figure 2D), preoperative Evan's index (*p* = 0.002), an extension of resection (*p* = 0.043) (Figure 2E), and preoperative CSF diversion (*p* = 0.024) (Figure 2F).

Considering tumor pathology, there is a statistically significant difference in developing postoperative hydrocephalus between medulloblastoma and teratoma (*p* = 0.033), ependymoma and AT/RT (*p* = 0.018), teratoma and AT/RT (*p* = 0.005), and teratoma and craniopharyngioma (*p* = 0.029) (Figure 2A). It also showed a statistically significant difference in leading to postoperative hydrocephalus between the fourth ventricle and cerebellopontine angle (CPA) (*p* = 0.008), and between sella and CPA (*p* = 0.021) when considering tumor location (Figure 2B). Taking into account the surgical approach, there is a statistically significant difference in developing persistent hydrocephalus between the posterior median approach and Poppen approach (*p* = 0.046), between the triangular approach and Poppen approach (*p* = 0.013), and between the Pterion approach and Poppen approach (*p* = 0.018) (Figure 2C). When aiming at preoperative Evan's index, which is a measure of the degree of ventricle dilatation, we found a significant difference between-group: 0.27–0.34 and group: 0.34–0.4 (*p* = 0.031), between-group: 0.27–0.34 and group: > 0.4 (*p* = 0.001) (Figure 2D). There is a statistically significant difference between gross total resection (>99%) and partial resection (50%-90%), and partial resection had a higher accidence of developing persistent hydrocephalus (Figure 2E). Tumors larger than 90cm^3^ were more likely to cause postoperative hydrocephalus than smaller tumors, which proved to be a statistically significant difference in our study (Table 2).

However, we did not find factors such as postoperative CSF diversion (*p* = 0.466), perioperative complication (hemorrhage or infection) (*p* = 0.167), and length of hospital stay (*p* = 0.713) were significantly correlated with persistent postoperative hydrocephalus. In addition, there were no statistically significant differences between different genders and between different ages (Table 2).

Notably, in the subgroup of children who underwent preoperative CSF diversion, we found that persistent postoperative hydrocephalus was significantly correlated with some kind of preoperative factors, such as tumor pathology (*p* = 0.040), tumor location (*p* = 0.014), preoperative Evan's index (*p* = 0.031), and tumor volume (*p* = 0.019) (Table 3). This result suggests these factors might impact the incidence of postoperative hydrocephalus in children who received CSF diversion before surgery. For tumors invading the fourth ventricle, it is more necessary to perform preoperative CSF diversion, either EVD, Ommaya reservoir, or VPS. We also found that preoperative Evan's index is significantly correlated with tumor volume (*p* = 0.019). A larger tumor volume suggested more severe ventriculomegaly (Table 4).

**Table 3:** Univariate analysis of the association between preoperative factors and persistent hydrocephalus in preoperative CSF diversion subgroup.

**Variables**	**Postoperative Hydrocephalus**		*P*-value^*^
	**Yes (*****n** =* **33)**	**No (*****n** =* **33)**	
**Tumor pathology**			^*^ **0.040** ^†^
Medulloblastoma	19 (57.6%)	11 (33.3%)	
Ependymoma	2 (6.1%)	4 (12.1%)	
Astrocytoma	4 (12.1%)	6 (18.2%)	
Teratoma	1 (3.0%)	6 (18.2%)	
Germinoma	0 (-)	3 (9.1%)	
Choroid plexus papilloma	0 (-)	1 (3.0%)	
Craniopharyngioma	1 (3.0%)	0 (-)	
Gangliogiloma or Oligodendroglioma	2 (6.1%)	2 (6.1%)	
Glioblastoma	1 (3.0%)	0 (-)	
AT/RT	3 (9.1%)	0 (-)	
**Tumor location**			^*^ **0.014** ^†^
Fourth ventricle	23 (69.7%)	13 (39.4%)	
Cerebellopontine angle	1 (3.0%)	8 (24.2%)	
Third ventricle/pineal gland	4 (12.1%)	9 (27.3%)	
Lateral ventricle	1 (3.0%)	2 (6.1%)	
Thalamus-basal ganglia	2 (6.1%)	1 (3.0%)	
Sella	1 (3.0%)	0 (-)	
Cerebral hemisphere	1 (3.0%)	0 (-)	
**Preoperative Evan's Index**			^*^ **0.031** ^‡^
0.27–0.34 (mild)	7 (21.9%)	18 (52.9%)	
0.34–0.4 (moderate)	14 (43.8%)	10 (27.4%)	
0.4 (severe)	11 (34.4%)	6 (17.6%)	
**Tumor volume (cm3)**			^*^ **0.019** ^†^
< 90	26 (42.6%)	35 (57.4%)	
>90	5 (100%)	0 (-)	

Values are reported as number (percentage). AT/RT, Atypical teratoid/rhabdoid tumor. ^*^Bold values are significant. ^†^Fisher exact test. ^‡^Pearson χ^2^.

**Table 4:** Association between preoperative Evan's Index and tumor volume.

**Evan's index**	**Tumor volume (cm^3^)**	*P* value^*^
0.27–0.34 (mild)	40.12 ± 36.26	^*^ **0.002** ^§^
0.34–0.4 (moderate)	47.25 ± 24.19	
>0.4 (severe)	118.49 ± 190.85	

Values are reported as mean ± SD. ^§^Student t-test.

^*^Bold values are significant.

### Univariate analysis in preoperative CSF diversion subgroup

Meanwhile, patients' need for preoperative CSF diversion was significantly correlated with tumor location (*p* = 0.019) (Table 5). Tumors that grew into the fourth and third ventricles were more likely to require preoperative hydrocephalus intervention than tumors in other locations, and such difference has been verified to be statistically significant in this cohort. However, the need for postoperative CSF diversion was not significantly correlated with all the factors mentioned above.

**Table 5:** Univariate analysis of the association between preoperative factors and Preoperative CSF diversion.

**Variables**	**Preoperative CSF diversion**		*P*-value^*^
	**Yes (*****n** =* **65)**	**No (*****n** =* **52)**	
**Tumor pathology**			0.065^†^
Medulloblastoma	30 (45.5%)	15 (29.4%)	
Ependymoma	6 (9.1%)	7 (13.7%)	
Astrocytoma	10 (15.2%)	12 (23.5%)	
Teratoma	7 (10.6%)	0 (-)	
Germinoma	3 (4.5%)	2 (3.9%)	
Choroid plexus papilloma	1 (1.5%)	3 (5.9%)	
Craniopharyngioma	1 (1.5%)	5 (9.8%)	
Gangliogiloma or Oligodendroglioma	4 (6.1%)	3 (5.9%)	
Glioblastoma	3 (4.5%)	3 (5.9%)	
AT/RT	1 (1.5%)	1 (2.0%)	
**Tumor location**			^*^ **0.019** ^†^
Fourth ventricle	36 (55.4%)	23 (44.2%)	
Cerebellopontine angle	9 (13.8%)	8 (15.4%)	
Third ventricle/pineal gland	13 (20.0%)	4 (7.7%)	
Lateral ventricle	3 (4.6%)	5 (9.6%)	
Thalamus-basal ganglia	3 (4.6%)	2 (3.8%	
Sella	0 (-)	4 (7.7%)	
Cerebral hemisphere	1 (1.5%)	6 (11.5%)	
**Preoperative Evan's Index**			0.815^‡^
0.27–0.34 (mild)	25 (37.9%)	22 (43.1%)	
0.34–0.4 (moderate)	24 (36.4%)	16 (31.4%)	
0.4 (severe)	17 (25.8%)	13 (25.5%)	
**Tumor volume (cm** ^3^ **)**			0.149^‡^
< 90	61 (92.4%)	42 (82.4%)	
>90	5 (7.6%)	9 (17.6%)	

Values are reported as number (percentage). AT/RT, Atypical teratoid/rhabdoid tumor. ^*^Bold values are significant. ^†^Fisher exact test. ^‡^Pearson χ^2^.

### Multivariate analysis identified the independent influencing factors

In the multivariate analysis, posterior median approach (*p* = 0.003; OR = 5.315; 95% CI 1.774–15.924), partial resection (*p* = 0.012, OR = 20.984, 95% CI 1.941–226.873), tumor volume larger than 90cm^3^ (*p* = 0.049, OR = 5.768, 95% CI: 0.983–33.856), and without preoperative CSF diversion (*p* = 0.009, OR = 3.661, 95% CI: 1.375–9.745) were confirmed as independent risk factors for persistent postoperative hydrocephalus, while Evan's index between 0.27 and 0.34 (*p* = 0.002, OR = 0.213, 95% CI: 0.081–0.559) predicted as a low-risk factor (Table 6).

**Table 6:** Multivariate analysis of factors associated with postoperative persistent hydrocephalus.

**Variables**	**Odds ratio (95% CI)**	***P*-value**
Posterior median approach	5.315 (1.774–15.924)	0.003
Partial resection	20.984 (1.941–226.873)	0.012
Volume >90 cm^3^	5.768 (0.983–38.856)	0.049
Evan's index (0.27–0.34)	0.213 (0.081–0.559)	0.002
No preoperative CSF diversion	3.661 (1.375–9.745)	0.009

## Discussion

There is still no consensus on the management of hydrocephalus before and after tumor resection (3, 7), especially in pediatric periventricular tumors. In the present study, we retrospectively analyzed clinical parameters, including sex, age, preoperative Evan's index, tumor pathology, volume and location, surgical approach, the extension of resection, and preoperative or postoperative diversion to identify whether these factors were correlated with persistent hydrocephalus after tumor resection.

Our results showed that posterior median approach, volume of > 90cm^3^, partial resection, and without preoperative CSF diversion were identified as significant risk factors for the development of postoperative hydrocephalus, while preoperative EI of < 0.34 was correlated with low incidence. Findings in this study may be used to preoperatively identify patients at high risk of postoperative hydrocephalus after resection of the childhood periventricular tumors and assess the necessity of preoperative CSF diversion.

In this study, we did not find age to be significantly associated with persistent hydrocephalus (Table 2). However, Bognar et al. (6) and Kumar et al. (16). reported that patients younger than 3 years old were at a higher rate of postoperative hydrocephalus, which might be explained by the finding that younger patients have an incompletely developed CSF absorption system (arachnoid granules, meningeal lymphatic vessels, and so on). Meanwhile, children at young ages are more likely to develop malignant tumors, such as medulloblastoma, high-grade glioma, and so on. Tumors above often obstruct the aqueduct at the first diagnosis, then recur in a short time after surgery. The discrepancy between our results and the results of previous studies may be because many older children also had developed large malignant midline tumors in this cohort, which affected the reliability of the analysis of age < 3 years as a predictive variable.

Riva-Cambrin et al. (8) demonstrated that, even though after adjusting for many other variables, medulloblastomas, ependymomas, and dorsally exophytic brainstem gliomas independently predisposed patients to persistent hydrocephalus. This study also demonstrated that tumor pathology was significantly correlated with the occurrence of persistent hydrocephalus. A significant difference in causing postoperative hydrocephalus was found between medulloblastoma and teratoma (*p* = 0.033), ependymoma and AT/RT (*p* = 0.018), teratoma and AT/RT (*p* = 0.005), and teratoma and craniopharyngioma (*p* = 0.029) (Figure 2A). However, in multiple analyses, we did not find medulloblastoma or ependymoma presented to be an independent risk factor for persistent postoperative hydrocephalus. The inconsistency between our results and those reported in the previous study may be that in addition to posterior fossa tumors, the present study includes many other types of periventricular tumors located in the CSF pathway and obstructed CSF flow, which increases the risk of postoperative hydrocephalus. Too few ependymoma cases in the cohort may also affect the accurate incidence.

Papo et al. (17) first suggested that a midline location near the fourth ventricle correlates with persistent hydrocephalus after excision, which also has been echoed in a retrospective study of 103 patients performed by Culley et al. (18) who found that those midline tumors were correlated with postoperative shunt placement. In this study, we found that tumor location was significantly associated with the occurrence of persistent hydrocephalus. Basically, 66.1% (39/59) of patients with tumors located in the fourth ventricle and 76.4% (13/17) of patients with tumors located in the third ventricle developed postoperative hydrocephalus after resection, compared with only 14.3% (1/7) of patients who had hydrocephalus occurring with tumors located in the cerebral hemisphere. In our study, more periventricular (not only the fourth but also third and lateral ventricles) tumors were included in the midline location, which may interfere with normal CSF flow and lead to post-resection ventriculitis. Notably, tumors located in the fourth ventricle and sella region were more likely to cause persistent postoperative hydrocephalus as per this study's findings (Figure 2B). Usually, tumors of the cerebral hemisphere, sellar region, and lateral anterior third ventricle may obliterate one site or both sites of the foremen of Monro. Tumors of the thalamus or basal ganglia would either block the foramen of Monro or plague the aqueduct. Tumors of the posterior fossa, including the cerebellar, fourth ventricle, and pons-medulla tumors can block the fourth ventricle (19).

The surgical approach was seldom considered an effective prediction for postoperative hydrocephalus. However, in our study, it was proven to be significantly correlated with persistent hydrocephalus after surgery. It was worth noting that in the multivariate analysis, the posterior median approach was considered to be an independent risk factor for postoperative persistent hydrocephalus. A possible explanation might be that the posterior median approach may lead to a stenosis of the CSF pathway (aqueduct and foramina of Luschka) (20), which is caused by postoperative hemorrhage, inflammatory adhesion, or unremoved residues of the tumor.

Unlike the previous study (18), we did not find a significant correlation between the diameter of the tumor of > 3cm and persistent postoperative hydrocephalus. However, to a certain extent, the tumor's size reflects the tumor's malignancy, determines the scope of resection, and then affects the occurrence of persistent hydrocephalus. Considering the importance of the size of children's periventricular tumors in predicting the risk of postoperative hydrocephalus, we took tumor volume into account. At last, we found that a volume of > 90 cm^3^ is a high-risk group for persistent hydrocephalus. In multivariate analysis, it is still considered an independent risk factor. A possible explanation might be tumors with larger volumes are more likely to be malignant and more prone to meningeal metastasis and recurrence. At the same time, the extensive growth of a giant tumor can increase the difficulties of completing resection, and the residual tumor after surgery promotes the development of persistent hydrocephalus.

Gross total resection of the tumor has been correlated with a low incidence of persistent hydrocephalus requiring shunt placement (21). Kumar et al. (16). found a significant difference in rates of shunt placement between patients that underwent gross total excision and those that had a partial resection (13% *vs*. 32%, respectively). Similar to a previous research, our study demonstrated that partial resection indicated a high risk of predicting postoperative hydrocephalus (Figure 2E). The residual tumor may obstruct the CSF flow through its mass effect, which means the cause of hydrocephalus has not been eliminated. However, some authors reasoned that total removal created a larger tumor bed followed by an intense CSF reaction resulting in hydrocephalus (22, 23).

There are conflicting reports as to whether the radiographic severity of hydrocephalus on initial clinical presentation is associated with persistent hydrocephalus. Riva-Cambrin et al. (8) and Tamburrini et al. (24) confirmed an association between quantitative moderate/severe preoperative ventriculomegaly (Evan's index of > 0.34) and persistent hydrocephalus. Similarly, in the present study, EI of > 0.34, defined as moderate and severe preoperative hydrocephalus, was considered a risk factor for persistent postoperative hydrocephalus. The higher the severity of hydrocephalus shown on imaging, the more likely patients developed persistent postoperative hydrocephalus (Figure 2D). This might be due to severe hydrocephalus causing increased venous and CSF pressure, and it may take a long time for the pressure to clear. Accompanied by resection of the tumor, an enlarged ventricle that has been adapted to the mass effect caused by the tumor may not be able to return to normal size for a long time after surgery. The multivariate analysis demonstrated that EI of < 0.34 was considered a prediction of the low incidence of persistent postoperative hydrocephalus.

As presented in our study, preoperative CSF diversion, including EVD, VPS, and Ommaya reservoir, could decrease the risk of persistent hydrocephalus. The preoperative CSF diversion in our study is a protective placement to relieve symptoms of intracranial hypertension and improve the general condition of children, and this is echoed in the studies by Tamburrini et al. (24) and Rappaport et al. (25). The authors favored preoperative EVD because it allowed for control of intracranial pressure and they believed that it correlated with a lower incidence of persistent hydrocephalus after tumor resection. EVD, VPS, and ETV are still considered effective measures to relieve symptoms caused by preoperative hydrocephalus before tumor resection and palliate difficulties of surgery. Temporary EVD is a practical approach to CSF diversion, which allows for continuous pressure monitoring, helps to remove surgical debris and blood products, and reduces the incidence of upward tentorial herniation or intratumoral hemorrhage (26). Preoperative endoscopic third ventriculostomy (ETV) is believed to reduce the incidence of postoperative hydrocephalus, has a lower complication rate, prevents excessive drainage of CSF, and is a faster operation compared with ventriculoperitoneal shunt replacement. However, we did not find that postoperative CSF management, especially VPS placement after resection, had a significant capacity to reduce the incidence of persistent postoperative hydrocephalus. As time goes by, the main strategy of tumor-associated hydrocephalus management in most institutes is primary tumor resection without a postoperative CSF shunt. CSF management needs to be taken into account before surgical resection rather than permanent shunt placement after surgery. On the other hand, cerebrospinal fluid shunts remain among the most failure-prone life-sustaining medical devices implanted in modern medical practice, with failure rates of 30–40% at 1 year and ~50% at 2 years in pediatric patients (27). Shunt malfunction (28) is an ongoing serious issue for pediatric neurosurgeons. Programmable shunts, which are considered to be an effective solution to shunt malfunction, have not reduced the shunt revision rate (29). Meanwhile, in this study, no evidence supported that there was a difference between EVD (58.6% without persistent hydrocephalus), VPS (33.3%), and Ommaya reservoir placement (45.4%) in reducing the incidence of postoperative persistent hydrocephalus. Similar results could be seen when concerning the subgroup of posterior fossa tumors. Recently, more and more studies reported (30, 31) that preoperative ETV reduced the incidence of persistent hydrocephalus and shunt placement postoperatively, which usually caused obstructive hydrocephalus at diagnosis (32). Furthermore, ETV is specially reserved for tumors of the thalamus, pineal region, tectum, and pons that obstruct the aqueduct or the fourth ventricle, and radical tumor resection is not considered (19). Ommaya reservoir, widely used in children's brain tumor-associated hydrocephalus in our institute, has several advantages such as low cost, immediate placement, low infection rate, low incidence of metastasis (33), and removability (34). More persuasive evidence (35) of the efficacy of the Ommaya reservoir in preoperative CSF diversion is still needed through practical research. In the future, a randomized trial in children with tumor-associated hydrocephalus is needed to compare the use of a preoperative ETV, VPS, EVD, or Ommaya reservoir.

This study also highlights the need for a multicenter trial to propose a predictive model that will help to guide the management of children with periventricular tumors. However, the present study has some limitations. As a single-center retrospective study, admission bias may be present in our sample, and this finding needs to be validated with prospective and large-scale studies.

## Conclusion

This study found that tumor pathology, tumor location, surgical approach, tumor resection degree, tumor volume, preoperative Evan's index, and preoperative CSF diversion were significantly correlated with persistent postoperative hydrocephalus in children with periventricular tumors. Notably, the posterior median approach, partial resection, a tumor volume larger than 90 cm^3^, and no preoperative CSF diversion were considered independent risk factors for persistent postoperative hydrocephalus, while an Evan's index of < 0.34 resulted as a significant protective factor. Prospective and larger scale studies are needed to verify this study.

## Data availability statement

The original contributions presented in the study are included in the article/supplementary material, further inquiries can be directed to the corresponding author.

## Ethics statement

The studies involving human participants were reviewed and approved by the Ethics Committee of Zhujiang Hospital. Written informed consent from the patients/participants or patients/participants' legal guardian/next of kin was not required to participate in this study in accordance with the national legislation and the institutional requirements.

## Author contributions

ZC: project design, formal analysis, investigation, data curation, visualization, writing—original draft, writing—review, and editing. MZ: project design, formal analysis, methodology, visualization, writing—original draft, writing—review, and editing. HW: project design, methodology, writing—review, and editing. JG: investigation and supervision. WZ: project administration, resources, supervision, writing—original draft, writing—review, and editing. All authors contributed to the article and approved the submitted version.
